# Absorption and Excretion of Antipyrine

**Published:** 1888-08

**Authors:** 


Absorption and Excretion of Antipyrine.—Dr. A. Mossella (*Gazetta degli Ospitali*, No. 73, 1887,) has employed antipyrine by the mouth, by the rectum, as an ointment, and hypodermically, and has followed up the excretion of it from the body. After the use of clysters (four parts of antipyrine dissolved in 100 of water), the temperature fell and excretion of antipyrine by the urine occurred. When employed externally, by means of painting with glycerine solutions, inunctions of solutions in almond oil into the arm and knee-joint, etc., no kind of absorption took place. After hypodermic injection into rabbits (one and a-half to one and three-quarter grains at a dose) antipyrine could be very quickly detected in the urine. On the other hand, after internal employment of the drug in pathological conditions, he was not successful in finding it in the sputum, pleuritic exudate, or echinococus cysts. Excretion through the saliva, the skin or the mammary glands could never be observed. Excretion seems to take place only through the urine.—*Deutsche Medicinal-Zeitung, March 26, 1888.*

